# The impact of destructive leadership on turnover intention among Chinese technology professionals: the mediating role of job burnout and the moderating role of regulatory emotional self-efficacy

**DOI:** 10.3389/fpsyg.2025.1698652

**Published:** 2025-11-17

**Authors:** Songqing Chen, Shuli Wang, Jun Wu

**Affiliations:** 1 Science and Technology Division, Yangzhou University, Yangzhou, China; 2 School of Chemistry and Chemical Engineering, Yangzhou University, Yangzhou, China

**Keywords:** destructive leadership, turnover intention, job burnout, regulatory emotional self-efficacy, job demands–resources theory, technology professionals, mediation, moderated direct effect

## Abstract

**Introduction:**

Destructive leadership is conceptualized as a social job demand that depletes employees’ psychological resources. Drawing on the Job Demands–Resources (JD–R) theory, we tested the mediating role of job burnout in the link between destructive leadership and turnover intention, and the moderating effect of regulatory emotional self-efficacy (RESE) on the direct path from destructive leadership to turnover intention.

**Methods:**

We analyzed survey data from 403 Chinese technology professionals using validated scales. Further, we tested whether job burnout mediates the link between destructive leadership and turnover intention, and whether RESE weakens the direct association between destructive leadership and turnover intention.

**Results:**

Destructive leadership and turnover intention were positively associated. Job burnout partially mediated this link (significant indirect effect), and RESE attenuated it; simple-slope tests revealed a weaker association for employees with higher RESE.

**Conclusion:**

The findings position destructive leadership as a resource-depleting social demand within JD–R, confirming burnout as a proximal mechanism linking it to turnover intention, and identifying RESE as a psychological buffer of the direct pathway. Organizations should deter destructive leadership and strengthen employees’ RESE to sustain wellbeing and mitigate talent loss in high-pressure technology settings.

## 1 Introduction

Rapid shifts in the technology industry have intensified competition for skilled professionals. A company’s recruitment and retention of technology professionals is pivotal to determining its competitive edge (Van Heerden et al., 2022). However, the industry’s high-intensity work pressure and management challenges have led to rising employee turnover rates, seriously threatening the stability and sustained innovation capabilities of businesses (Tomer et al., 2022). To effectively address this challenge, enterprises must understand the specific motivations driving technology professionals’ decisions to resign, so that they can retain core professionals and maintain a competitive advantage.

The factors influencing the decision of technology professionals to resign are diverse and multifaceted, encompassing a range of dimensions (Wang et al., 2021; Sun et al., 2022; Jogi et al., 2025). They include compensation and benefits, career development opportunities, work pressure, management styles, and leadership approaches. Among these, destructive leadership behaviors (e.g., abusive management, emotional suppression, authoritarian decision-making, and personal degradation) have attracted increasing attention within academic and professional circles due to their substantial negative impact on employees’ psychological wellbeing and behavior (Li et al., 2024). While research has indicated that destructive leadership significantly increases employees’ turnover intention (Calderon-Mafud et al., 2024), within the context of China’s technology industry, characterized by high-pressure work environments, fast-paced work rhythms, and high demands for innovation, the specific mediating mechanisms of this relationship and the boundary conditions for its effects remain underexplored.

This study’s objective is to address two significant research gaps within the Job Demands-Resources (JD–R) theoretical framework (Bakker and Demerouti, 2024). First, the present study examines the mediating role of job burnout (Maslach et al., 2001) in the relationship between destructive leadership and turnover intention. In light of the technology industry’s high-pressure environment, work burnout is regarded as a crucial psychological mechanism that elucidates this influence process. Second, the study investigates the moderating effect of regulatory emotional self-efficacy (RESE) (Li and Quan, 2025), a pivotal individual psychological resource (job resource). RESE refers to an individual’s belief in their ability to manage negative emotions (e.g., anger and frustration) and stimulate positive ones. This ability is critical in dealing with negative emotional experiences triggered by destructive leadership. However, the extent to which it fulfills a buffering role in the chain of effects of destructive leadership remains to be fully elucidated through empirical testing.

The present study makes theoretical and practical contributions in the following areas. First, it contributes to the JD–R theory by framing destructive leadership as a type of socially demanding job requirement that continuously depletes employees’ emotional and psychological resources (e.g., patience, self-confidence, and emotional stability). While prior research on JD–R theory has predominantly centered on the impact of task-related demands (Chowhan and Pike, 2023), such as workload and organizational resources, including social support, it has neglected to consider the significant influence of leadership as a social factor on employees’ mental and physical health and behavioral intentions. Consequently, the present study broadens the application scope of the JD–R theory in the context of the relationship between leadership behavior and employee mental health by investigating the influence pathways of destructive leadership as a consumptive social job demand on employees’ turnover intentions.

Second, this study elucidates how work burnout acts as a mediator in the relationship between destructive leadership and employees’ intention to leave. Burnout is a negative psychological state that not only severely damages employees’ work enthusiasm and efficiency but also leads to the development of intense feelings of detachment and rejection toward their current position and even the organization. In the context of technology companies, where workloads are notoriously high, the deleterious effects of destructive leadership are exacerbated. The incessant exhaustion of employees’ psychological resources frequently results in burnout, reinforcing their inclination to resign (Rosa et al., 2024). By elucidating this mediating pathway, the present study offers a concrete and compelling psychological explanatory framework for understanding talent loss in the technology industry.

Third, the study introduces the individual psychological resource of regulatory emotional self-efficacy (RESE), exploring its protective role against the negative effects of destructive leadership. RESE has been demonstrated to reflect employees’ ability to cope with negative emotions and adapt to adversity, serving as a buffer in mitigating the psychological toll caused by destructive leadership (Brecciaroli et al., 2024). Despite previous studies’ recognition of the significance of individual psychological resources within the JD–R theoretical framework, empirical research on the mechanisms by which RESE moderates the effect on the relationship between destructive leadership and turnover intention remains limited. The present study addresses this gap by examining RESE’s moderating role in the influence pathways of destructive leadership on work burnout and subsequent turnover intentions, clarifying the important boundary conditions under which RESE serves as a buffer against negative leadership behaviors.

The research context for this study is Chinese technology industry, distinguished by its high work intensity, rapid innovation pace, and frequent talent turnover. Cultural factors, such as high-power distance and a strong collectivist orientation, have the potential to further amplify the impact of leadership behavior on employee psychology. This study, therefore, addresses the current academic interest in the differential effects of destructive leadership behavior across different cultural and industrial contexts, contributing to the theoretical body of knowledge on this type of leadership by offering localized theoretical foundations and practical insights for Chinese technology company managers. These insights can facilitate the development of effective intervention measures, the optimization of leadership behavior patterns, and the reduction of employee turnover rates.

In summary, this study integrates destructive leadership, job burnout, and RESE to construct and assess a mediation model with a moderated direct destructive leadership–turnover intention association. This model aims to elucidate the influence of destructive leadership on employees’ intention to resign through the psychological resource depletion pathway. Additionally, it seeks to clarify the specific moderating role of RESE in this process. Through this exploration, the study is expected to deepen the JD–R theory, expand the theoretical boundaries of destructive leadership research, and provide policy implications and feasible, practical guidance for optimizing leadership behavior and protecting employee mental health.

## 2 Theoretical framework and hypotheses

### 2.1 Theoretical background: job demands-resources theory

The Job Demands-Resources theory posits that working conditions cluster into job demands, aspects of the job that require sustained physical or psychological effort, and job resources, aspects that help achieve work goals, stimulate growth, and reduce demands (Demerouti et al., 2001; Bakker and Demerouti, 2017, 2024). Persistent, high-intensity job demands, for example work overload, interpersonal conflict, and role ambiguity, trigger a health-impairment process that progressively depletes employees’ energetic and psychological resources and fosters burnout. Conversely, job resources fuel a motivational process and can buffer the deleterious impact of demands (Bakker et al., 2005; Arshad et al., 2020; Mohamad et al., 2025). Within this framework, personal resources, such as self-efficacy, are integral to how individuals interpret and cope with demands and may operate as moderators or mediators in JD–R processes (Xanthopoulou et al., 2007).

Building on JD–R and Conservation of Resources (COR) theory, which asserts that stress results from actual or threatened resource loss (Hobfoll, 1989), we conceptualize destructive leadership as a chronic social job demand. Destructive leader behaviors, for example abusive supervision, hostile communication, and authoritarian decision-making, undermine subordinates’ socioemotional functioning, erode resources, and heighten strain (Tepper, 2000; Schyns and Schilling, 2013; Li et al., 2024). These behaviors: (a) provoke negative affect, including anxiety, anger, and frustration, which accelerates resource loss spirals according to COR; (b) disrupt positive social exchange and recognition, which reduces access to instrumental and emotional job resources; and (c) increase role ambiguity and role conflict and uncertainty, which together maintain employees in a high-stress state (Bakker and Demerouti, 2017; Fischer et al., 2021).

Consistent with the health-impairment process, job burnout, classically comprising emotional exhaustion, cynicism, and reduced professional efficacy, emerges as a core psychological mechanism linking destructive leadership to adverse outcomes (Maslach et al., 2001). Meta-analytic evidence indicates that heightened demands and depleted resources robustly predict burnout, which in turn relates to turnover intention (Alarcon, 2011). Turnover intention (TI), employees’ deliberated propensity to leave, has long been recognized as a proximal predictor of actual turnover in organizational research (Tett and Meyer, 1993), and is therefore a theoretically appropriate focal outcome in our model.

We further introduce regulatory emotional self-efficacy (RESE) as a key personal resource that delineates boundary conditions in the above processes. RESE reflects confidence in one’s ability to manage negative emotions and to express positive emotions (Caprara et al., 2008). In JD–R terms, higher RESE equips employees with stronger self-regulatory capacity to cope with demand-induced affect, thereby weakening the linkage from destructive leadership to burnout. Recent evidence in high-strain settings supports RESE’s buffering role vis-à-vis workload-related exhaustion and interpersonal strain (Brecciaroli et al., 2024), and Frontiers-published work similarly shows resources mitigating the sequence from demand to burnout to turnover (e.g., Sun et al., 2025).

In sum, anchored in JD–R and COR, we conceptualize destructive leadership as a social job demand that increases turnover intention via job burnout (mediation), and we posit that RESE—as a personal resource—buffers the direct destructive leadership–turnover intention association. This logic underpins our conceptual framework and the hypotheses tested in the present study.

### 2.2 Hypotheses development

#### 2.2.1 Destructive leadership and turnover intention

Destructive leadership (DL) refers to leaders’ systematic and repeated behaviors that violate legitimate organizational interests and undermine followers’ motivation and wellbeing (e.g., hostile communication, abuse of power, excessive control) (Einarsen et al., 2007; Krasikova et al., 2013). From the perspective of social exchange theory, such behaviors erode reciprocity norms that sustain high-quality leader–member relationships; as reciprocity is undermined, employees are more likely to withdraw and contemplate leaving (Blau, 1964; Cropanzano and Mitchell, 2005).

A substantial body of meta-analytic evidence indicates small-to-moderate positive associations between destructive/abusive leadership and employees’ turnover intention (Schyns and Schilling, 2013; Mackey et al., 2017). In addition, a longitudinal meta-analysis further shows lagged adverse effects on employee attitudes and behaviors (Li et al., 2024). Classic primary studies converge on this pattern, showing that exposure to abusive supervision predicts stronger quitting tendencies (Tepper, 2000).

Within the Job Demands–Resources framework, destructive leadership can be conceptualized as a chronic social job demand that depletes emotional and cognitive resources—via heightened role ambiguity, negative affect, and psychological insecurity—thereby increasing withdrawal cognitions (Bakker and Demerouti, 2007, 2017, 2024). In technology-intensive settings characterized by fast innovation cycles and sustained pressure, such resource depletion may be especially consequential for knowledge workers’ retention decisions; evidence from Chinese samples likewise links abusive supervision to higher turnover intention (Wang et al., 2021).

Hypothesis 1 (H1): Destructive leadership is positively associated with Chinese technology professionals’ turnover intention.

#### 2.2.2 The mediating role of job burnout

Job burnout (JB) is a chronic, work-related strain syndrome comprising emotional exhaustion, cynicism, and reduced professional efficacy (Maslach et al., 2001). Within the Job Demands–Resources (JD–R) framework, sustained job demands initiate a health-impairment process that depletes energetic and psychological resources and elevates job burnout, whereas job resources buffer this process (Bakker and Demerouti, 2017; Lesener et al., 2019). Meta-analytic evidence further indicates that job burnout—especially emotional exhaustion—shows robust positive associations with turnover intention, clarifying why resource-depleting environments translate into quitting cognitions (Lee and Ashforth, 1996; Swider and Zimmerman, 2010; Alarcon, 2011).

Positioning destructive leadership (DL) as a social/interpersonal job demand, prior meta-analyses show that abusive/destructive leader behaviors reliably predict employee strain outcomes including emotional exhaustion and job burnout (Schyns and Schilling, 2013; Mackey et al., 2017). Longitudinal evidence further corroborates that DL prospectively undermines employees’ wellbeing and attitudes, strengthening inference beyond cross-sectional designs (Li et al., 2024; Malik et al., 2023). Thus, we hypothesize:

*H2*: Destructive leadership is significantly and positively associated with job burnout.

Building on JD–R, job burnout is expected to serve as the key psychological mechanism linking DL to turnover intention. JD–R specifies that job demands first impair health (i.e., elevate job burnout) and subsequently shape work attitudes and behaviors (Bakker and Demerouti, 2017; Lesener et al., 2019). Empirically, studies document that emotional exhaustion/job burnout mediates the effects of abusive/destructive supervision on turnover intention, aligning with the JD–R health-impairment path (Ali et al., 2022). Therefore:

*H3*: Job burnout mediates the relationship between destructive leadership and turnover intention.

#### 2.2.3 The moderating role of regulatory emotional self-efficacy

Regulatory Emotional Self-Efficacy (RESE) denotes individuals’ beliefs in their capability to manage negative emotions and to express positive affect when facing challenges. Conceptually and operationally, RESE captures perceived capacity for adaptive emotion regulation at work, which positions it as a personal resource relevant to stressor–outcome processes (Caprara et al., 2008).

Within Job Demands–Resources theory, personal resources such as self-efficacy help employees withstand high job demands by preventing energy loss and safeguarding wellbeing; consequently they can buffer the demands–strain and demands–attitudes pathways (Xanthopoulou et al., 2007; Bakker and Demerouti, 2017; Bakker et al., 2023). Leadership behaviors are integral to this context and can operate as job demands when they are destructive or abusive, thereby elevating strain and withdrawal cognitions (Tummers et al., 2021). From a Conservation of Resources perspective, RESE also functions as a resource that mitigates the deleterious consequences of events that threaten valued resources (Hobfoll, 1989).

A growing body of research links RESE, or closely related emotion-regulation efficacy, to lower burnout and better adjustment. Efficacy in managing negative emotions predicts reduced burnout (Alessandri et al., 2018), and meta-analytic findings indicate an inverse association between self-efficacy and burnout (Shoji et al., 2016). Recent field evidence further shows that RESE attenuates stressor–exhaustion linkages, for example workload–exhaustion, which is consistent with a buffering role in Job Demands–Resources processes (Brecciaroli et al., 2024).

RESE is also consequential for retention-relevant attitudes. A longitudinal three-wave study reported that RESE reduces turnover intentions indirectly by fostering organizational socialization and identification (Cepale et al., 2021). Moreover, in the destructive and abusive leadership domain, employees’ emotion-regulation capabilities and strategies have been shown to moderate the adverse effects of abusive supervision on strain and withdrawal, which supports the boundary-condition logic for RESE (Chi and Liang, 2013).

Taken together, when destructive leadership is conceptualized as a social and interpersonal job demand, employees with higher RESE should be better equipped to down-regulate negative affect and to maintain adaptive functioning, thereby weakening the translation of destructive leadership into turnover cognitions.

*H4*: Regulatory Emotional Self-Efficacy buffers the positive association between destructive leadership and turnover intention such that this association is weaker at higher levels of RESE.

### 2.3 Theoretical model

**FIGURE 1:**
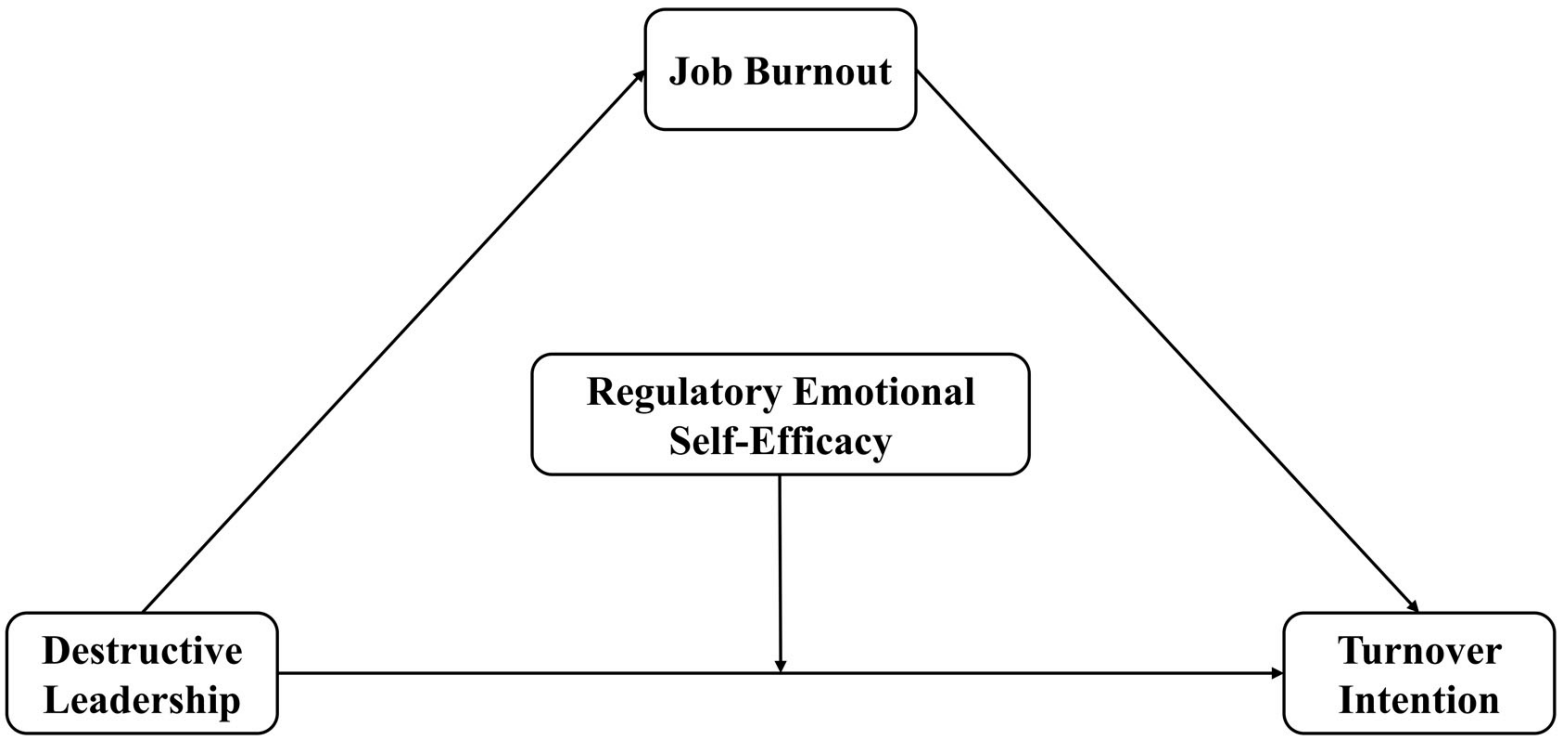
Research model.

Based on the theoretical derivations and assumptions detailed above, this study proposes a mediation model in which regulatory emotional self-efficacy moderates the direct path from destructive leadership to turnover intention. Within the Job Demands–Resources theory, destructive leadership is conceptualized as a social job demand that heightens turnover intention both directly and indirectly through job burnout. Regulatory emotional self-efficacy is treated as a personal resource that buffers the direct association between destructive leadership and turnover intention, such that higher regulatory emotional self-efficacy weakens this association. Figure 1 illustrates the theoretical model.

## 3 Materials and methods

### 3.1 Participants

We recruited full-time technology professionals employed in China. Eligibility criteria for inclusion were: (i) age ≥ 18; (ii) current employment within a technology-intensive sector, such as software and information technology, electronics, or advanced manufacturing; (iii) a minimum tenure of ≥ 6 months under the current supervisor; and (iv) the ability to read Chinese. Interns, currently unemployed individuals, and those working in non-technical/administrative roles were excluded. Sample characteristics and descriptive statistics are summarized in Table 1.

**TABLE 1:** Demographic information of respondents.

Variable	Item	Frequency	Percent (%)
Gender	Male	199	49.38
	Female	204	50.62
Age	≤ 25	45	11.17
	26–30	150	37.22
	31–35	124	30.77
	36–40	49	12.16
	≥ 41	35	8.68
Education level	College degree and below	67	16.63
	Bachelor degree	177	43.92
	Master’s degree	94	23.33
	Doctor’s degree	65	16.13
Work seniority	≤ 1	31	7.69
	1–5	185	45.92
	6–10	139	34.49
	≥ 10	48	11.91

### 3.2 Procedure

We employed a multi-channel, non-probability approach for recruitment. The survey link was disseminated via WeChat, QQ, and email. Data were collected between May and June 2024. A single reminder was sent approximately 1 week after the initial invitation. To minimize the number of duplicate submissions, responses were restricted to one per device/IP address. Before starting the questionnaire, participants read an electronic informed-consent statement describing the study purpose, clarifying the voluntary nature of their participation, guaranteeing their anonymity, and detailing the estimated completion time and the data-use policy. The study was conducted in accordance with applicable ethical standards and relevant local legislation, and electronic informed consent was obtained from all participants prior to data collection. We distributed 450 invitations and retained 403 valid responses after quality screening (valid response rate: 89.56%).

### 3.3 Measures

#### 3.3.1 Destructive leadership

Destructive leadership was measured using a five-item scale developed by Mitchell and Ambrose (2007), originally derived from Tepper (2000) abusive supervision measure. A sample item is “My supervisor tells me I’m incompetent.” Items were rated from 1 = “strongly disagree” to 5 = “strongly agree.” For language adaptation, items underwent forward–back translation by two independent bilingual experts, reconciliation by a third expert, and cognitive pretesting (*n* = 30). Cronbach’s alpha was 0.868.

#### 3.3.2 Job burnout

Job burnout was measured using the 15-item Chinese scale developed by Li and Shi (2003). A sample item from this scale is “Working all day is really a strain for me.” Items were assessed on a 5-point Likert scale from 1 = “strongly disagree” to 5 = “strongly agree.” The instrument comprises three dimensions: Emotional Exhaustion (EX; items 1–5), Cynicism (CY; items 6–9), and Professional Efficacy (PE; items 10–15).

Job Burnout scoring: For a 1–5 scale, reverse-code each Professional Efficacy item (items 10–15) as item* = 6 - item. Compute dimension means EX̄ = mean (items 1–5), CȲ = mean (items 6–9), and PĒ* = mean(reverse-coded items 10–15). Composite burnout = mean(EX̄, CȲ, PĒ*). Higher scores indicate higher burnout. The Cronbach’s alpha for this study was 0.943.

#### 3.3.3 Regulatory emotional self-efficacy

RESE was measured using the 12-item scale by Caprara et al. (2008), e.g., “Keep from getting discouraged by strong criticism?” Items were rated from 1 = “very poor ability” to 5 = “strong ability.” For language adaptation, items underwent forward–back translation (two independent bilingual translators + reconciliation) and cognitive pretesting (*n* = 30). Studies in Chinese samples have reported reliable psychometrics for Chinese versions of RESE (e.g., Wen et al., 2009; Zhang et al., 2022). Cronbach’s alpha in this study was 0.946.

#### 3.3.4 Turnover intention

Turnover intention was measured using the four-item Chinese scale developed by Weng and Xi (2010). The scale included items such as “I often feel bored with my current job and want to change to a new organization,” rated from 1 = “strongly disagree” to 5 = “strongly agree.”

Turnover Intention scoring: Let TI1–TI4 denote the four items; TI1 and TI2 are reverse-scored. For a 1–5 scale, compute TI1* = 6 - TI1 and TI2* = 6 - TI2; keep TI3 and TI4 unchanged. Composite turnover intention = mean (TI1*, TI2*, TI3, TI4). Higher scores indicate stronger turnover intention. The Cronbach’s alpha for this study was 0.863.

### 3.4 Data analyses

We conducted the confirmatory factor analysis (CFA) in AMOS 26 to assess the distinctiveness and validity of the four constructs (standardized loadings > 0.60; composite reliability > 0.70; average variance extracted > 0.50; model fit indices reported in Table 2). The mediation effect was tested using PROCESS Model 4 with 5,000 percentile bootstrap resamples (bias-corrected 95% CIs). A moderated direct effect was examined using PROCESS Model 5 with RESE as the moderator, with simple slopes probed at ± 1 SD. Analyses were conducted in SPSS 26, including gender and age as covariates. Data quality checks included removing speeders (completion time < one-third of the median), straight-lining/long strings (within-scale SD < 0.10), duplicate device/IP entries, and cases with excessive missingness (items with < 5% missing retained).

**TABLE 2:** Confirmatory factor analysis results.

Variable	AVE	CR	Cronbach’s α
Destructive leadership	0.567	0.868	0.868
Job burnout	0.527	0.943	0.943
Regulatory emotional self-efficacy	0.596	0.946	0.946
Turnover intention	0.613	0.864	0.863

We assessed non-response bias using a wave-analysis proxy, comparing early (first quartile) versus late (last quartile) respondents based on submission order. Welch tests on the four focal constructs showed no material differences (DL: *p* = 0.53; JB: *p* = 0.46; RESE: *p* = 0.92; TI: *p* = 0.45), and chi-square tests on available demographics indicated no systematic early–late differences. These results suggest that non-response bias is unlikely to threaten inference.

A *post hoc* sensitivity analysis (α = 0.05, 80% power; *N* = 403) indicated that the study had adequate power to detect small effects typical of field research. For mediation and moderation, we used 5,000 percentile bootstrap resamples and report 95% confidence intervals (CIs). Consistent with this approach, the DL–JB–TI indirect effect was 0.061, 95% CI (0.029, 0.088).

As well as the main OLS/PROCESS estimates, we conducted robustness checks with HC3 heteroskedasticity-robust standard errors; this confirmed that the conclusions remained unchanged. We report 95% CIs for all key effects and summarize basic assumption checks (linearity, low multicollinearity, and residual patterns). Discriminant validity was established using the Fornell–Larcker criterion: for example, the square root of the AVE for RESE (0.772) exceeded the absolute inter-construct correlation with turnover intention (0.538).

## 4 Results

### 4.1 Common method variance diagnostics

Harman’s single-factor test (unrotated) across all substantive items (Q5–Q40; *N* = 403) indicated that the first factor accounted for 33.64% of the variance, which is below the conventional 50% threshold for serious common method variance (CMV) concerns (Podsakoff et al., 2003). In addition, full collinearity variance inflation factors (Kock, 2015) based on construct composites were DL = 1.32, JB = 1.17, RESE = 1.49, TI = 1.65 (all < 3.3), suggesting that CMV is unlikely to materially bias the estimates.

### 4.2 Confirmatory factor analysis

CFA supported a four-factor measurement model comprising destructive leadership (DL; 5 items), job burnout (JB; 15 items), regulatory emotional self-efficacy (RESE; 12 items), and turnover intention (TI; 4 items). The model demonstrated excellent global fit, χ^2^(588) = 613.152, *p* = 0.229, χ^2^/df = 1.043, CFI = 0.997, TLI = 0.997, RMSEA = 0.010 with 90% CI (0.009, 0.019), and SRMR = 0.031 (GFI = 0.924, AGFI = 0.914, NFI = 0.933, IFI = 0.997 are reported for completeness). All standardized loadings were significant (all *p*s < 0.001) and fell within acceptable ranges (DL: 0.729in accepall ess). All tention (TI; 4 items). The model demonstrated excellent global fit, ≥ 0.70; AVE ≥ 0.50), with construct-level CR, AVE, and Cronbachpalα reported in Table 2 (DL: CR = 0.868, AVE = 0.567; JB: CR = 0.943, AVE = 0.527; RESE: CR = 0.946, AVE = 0.596; TI: CR = 0.864, AVE = 0.613). Discriminant validity was evidenced by the Fornell–Larcker criterion—the square roots of AVE (DL = 0.753; JB = 0.726; RESE = 0.772; TI = 0.783) exceeded the largest absolute inter-construct correlation (|r| ≤ 0.538, between RESE and TI) absoluteTMT values that fell below the conservative 0.85 threshold (max HTMT = 0.595). Collectively, these results indicate satisfactory measurement properties and justify proceeding to the structural analyses.

### 4.3 Correlation analysis

Table 3 provides a detailed overview of the descriptive statistics and correlations among the studied variables. The results reveal a positive correlation between destructive leadership and turnover intention (r = 0.460, *p* < 0.01), as well as between destructive leadership and job burnout (*r* = 0.211, *p* < 0.01). Similarly, job burnout is positively correlated with turnover intention (*r* = 0.350, *p* < 0.01). Furthermore, there is a negative correlation between RESE and turnover intention (*r* = –0.538, *p* < 0.01).

**TABLE 3:** Means, standard deviations, and Pearson correlations.

Variable	Mean	SD	1	2	3	4
1. Destructive leadership	2.284	1.021	1			
2. Job burnout	2.061	0.832	0.211**	1		
3. Regulatory emotional self-efficacy	3.638	1.015	–0.389**	–0.302**	1	
4. Turnover intention	2.421	1.103	0.460**	0.350**	–0.538**	1

*p < 0.05, **p < 0.01.

### 4.4 Mediation analysis

We utilized Model 4 in the SPSS macro-PROCESS to examine the potential impact of destructive leadership on turnover intention, with job burnout serving as the mediator. As demonstrated in Table 4, destructive leadership was positively correlated with both turnover intention (β = 0.459, *p* < 0.01) and job burnout (β = 0.211, *p* < 0.01). Furthermore, job burnout was positively correlated with turnover intention (β = 0.265, *p* < 0.01). Subsequent analysis revealed that destructive leadership exerts a significant influence on turnover intention through the mediating effect of job burnout. This was evidenced by the bias-corrected indirect effect test, which showed an indirect effect of 0.061 (SE = 0.015) with a 95% confidence interval of (0.029, 0.088). The mediating effect accounted for 12.23% of the total effect between destructive leadership and turnover intention. These results support H1 (DL–TI) and H2 (DL–JB). The significant indirect effect [0.061, 95% CI (0.029, 0.088)] further supports H3, indicating that job burnout partially mediates the DL–TI association.

**TABLE 4:** Results of mediating effect analysis.

	Turnover intention					Job burnout					Turnover intention				
	B	SE	*t*	*p*	β	B	SE	*t*	*p*	β	B	SE	*t*	*p*	β
Constant	1.348**	0.237	5.694	0.000	–	1.661**	0.197	8.434	0.000	–	0.764**	0.246	3.107	0.002	–
Gender	–0.074	0.098	–0.752	0.452	–0.034	0.011	0.082	0.132	0.895	0.007	–0.078	0.094	–0.826	0.409	–0.035
Age	0.020	0.045	0.437	0.662	0.020	–0.004	0.037	–0.098	0.922	–0.005	0.021	0.043	0.486	0.627	0.021
Destructive leadership	0.495**	0.048	10.327	0.000	0.459	0.172**	0.040	4.320	0.000	0.211	0.435**	0.047	9.252	0.000	0.403
Job burnout											0.351**	0.058	6.098	0.000	0.265
*R* ^2^	0.213					0.045					0.280				
*R*^2^ adjust	0.207					0.038					0.273				
*F*	*F*(3, 399) = 36.029, *p* = 0.000					*F*(3, 399) = 6.223, *p* = 0.000					*F*(4, 398) = 38.767, *p* = 0.000				

*p < 0.05, **p < 0.01.

### 4.5 Moderation analysis

**FIGURE 2:**
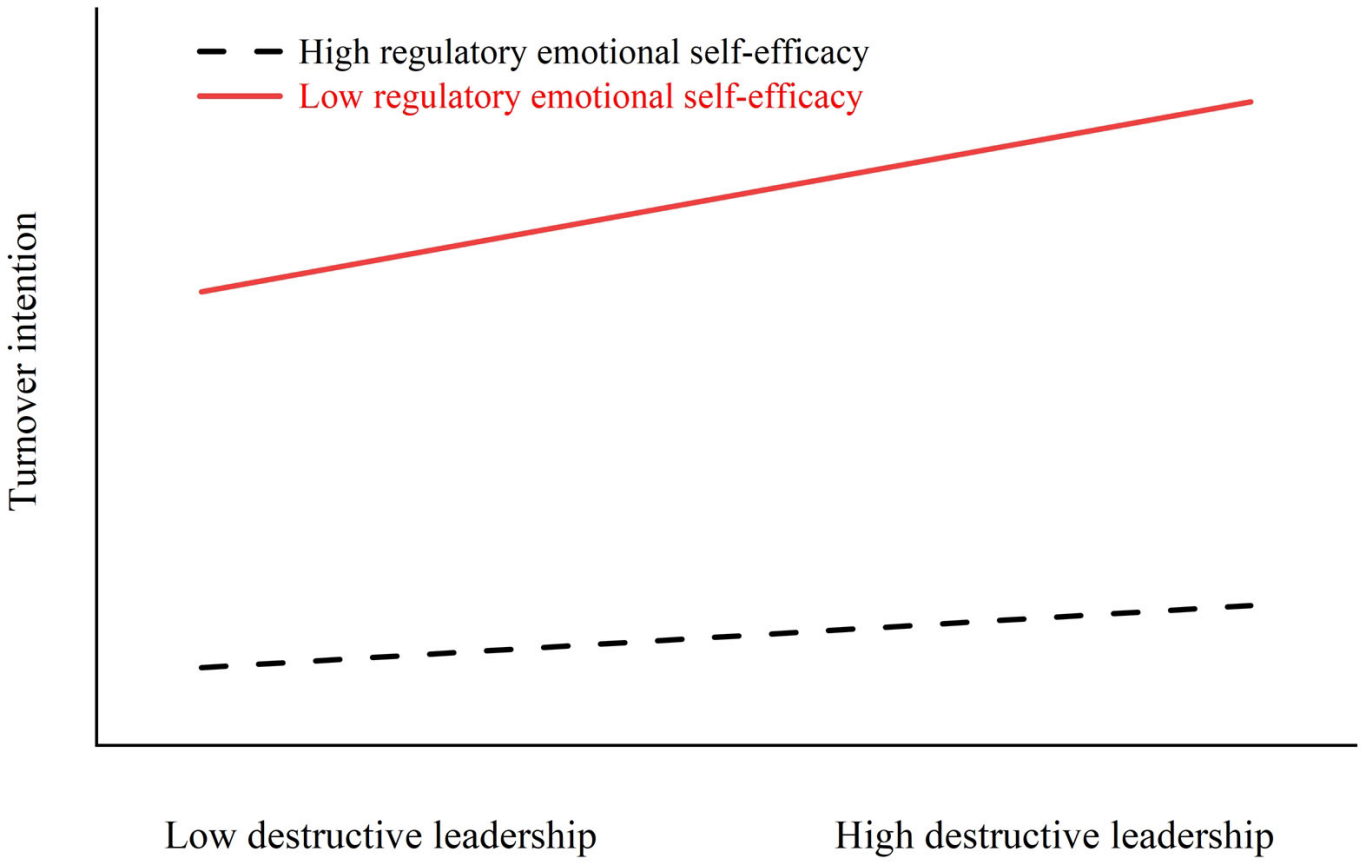
The moderating effect of regulatory emotional self-efficacy on the relationship between destructive leadership and turnover intention.

We tested the moderating effect of RESE on the direct path between DL and TI. The DL × RESE interaction was negative and statistically significant (Table 5), indicating that higher levels of RESE weaken the positive association between destructive leadership and turnover intention. Simple slope analyses showed that when RESE was low, DL was a significantly stronger predictor of TI [*B* = 0.485, 95% CI (0.355, 0.616)]; when RESE was high, the slope was significantly weaker [*B* = 0.165, 95% CI (0.040, 0.291)]. Both conditional slopes were positive, consistent with a buffering pattern rather than a crossover effect. The visualization presented in Figure 2 mirrors these results, where the low-RESE line is markedly steeper than the high-RESE line across the observed range of DL.

**TABLE 5:** Results of the moderation model.

Moderator	Simple slope	SE	95%CI	
			Lower	Upper
High regulatory emotional self-efficacy	0.165	0.067	0.040	0.291
Low regulatory emotional self-efficacy	0.485	0.064	0.355	0.616

## 5 Discussion

Consistent with the Job Demands–Resources (JD–R) framework, the present study found a positive association between destructive leadership (DL) and turnover intention (TI). In the baseline model, DL demonstrated a substantial total effect on TI (*B* = 0.495, *p* < 0.001). After introducing job burnout (JB) to the model, the direct effect of DL on TI remained significant; however had decreased (*B* = 0.435, *p* < 0.001), indicating partial mediation.

The mediating role of JB in the link between DL and TI was statistically supported, aligning with the JD–R health-impairment process. The bias-corrected bootstrap indirect effect of DL on TI via JB was 0.061 [95% CI (0.029, 0.088)], accounting for 12.23% of the total effect. This pattern suggests that the social/interpersonal demands placed on employees by DL deplete their resources, heighten strain, and ultimately lead to withdrawal cognitions.

The moderation analysis revealed that RESE buffered the DL–TI association. Simple-slope probes showed that the DL–TI slope was stronger at low RESE (*B* = 0.485, 95% CI (0.355, 0.616)] and weaker at high RESE [*B* = 0.165, 95% CI (0.040, 0.291)]. Both slopes were positive, illustrating the detrimental effect of DL across RESE levels; however, the smaller slope at high RESE clearly demonstrates a pattern of attenuation (buffering) rather than a crossover effect.

Collectively, the results support all focal hypotheses, confirming that DL is positively associated with TI (H1), that JB partially transmits the effect of DL on TI (partial mediation) (H2–H3), and that RESE weakens the direct link between DL and TI (H4). The integrated evidence identifies DL as a chronic social demand that erodes resources and increases burnout, while RESE—an individual resource—mitigates the translation of DL into quitting cognitions.

### 5.1 Theoretical implications

This study advances organizational behavior theory by extending the JD–R framework to explicitly treat DL as a social job demand. While prior applications of the JD–R theory have emphasized task demands (e.g., workload) and structural resources, we conceptualize DL as a persistent interpersonal stressor that erodes employees’ resources through the health-impairment process (Demerouti et al., 2001; Bakker and Demerouti, 2017). This framing aligns with leadership research that defines DL as systematic, repeated behavior that undermines follower well-being and organizational goals (Einarsen et al., 2007) as well as with meta-analytic evidence of robust associations between destructive or abusive leadership and adverse employee outcomes (Schyns and Schilling, 2013; Mackey et al., 2017).

Second, our findings clarify the psychological mechanism by which DL influences turnover intentions by identifying JB as a key mediator. This aligns with evidence establishing a reliable link between burnout—especially emotional exhaustion and cynicism—and withdrawal cognitions and intentions to quit (Swider and Zimmerman, 2010; Chen et al., 2024). By understanding burnout as the conduit through which social demands deplete resources, our study integrates leadership research with JD–R’s health-impairment pathway to help explain why negative leadership behaviors propagate adverse organizational outcomes (Bakker and Demerouti, 2017; Wei et al., 2025).

Third, by introducing RESE as a moderator, we delineate the boundary conditions under which DL translates into burnout and turnover intentions. RESE, which captures an individual’s perceived capability to manage negative affect and express positive affect (Caprara et al., 2008), falls within the wider category of personal resources emphasized in JD–R (Xanthopoulou et al., 2007). Our results are therefore consistent with the Conservation of Resources theory, which posits that resource possession reduces vulnerability to loss and buffers stress responses (Hobfoll et al., 2018). Individuals with higher RESE appear to exhibit a reduced vulnerability to the emotional damage induced by DL, attenuating its downstream effects on burnout and turnover intentions.

### 5.2 Practical implications

Our findings suggest that organizations—particularly those in high-stress, technology-intensive contexts—should establish robust mechanisms for identifying, managing, and remediating destructive leadership practices. This recommendation is also supported by meta-analytic evidence linking destructive and abusive leadership to a wide range of adverse employee outcomes (Schyns and Schilling, 2013; Mackey et al., 2017). Additionally, JD–R theory predicts that reducing hindrance-type social demands helps curb health impairment processes (Demerouti et al., 2001; Bakker and Demerouti, 2017). Translationally, this suggests the need for organizations to introduce leadership standards, 360-degree feedback that is tied to performance, and targeted coaching for at-risk managers, alongside clear reporting and remediation protocols.

Second, identifying JB as a mediator underscores the importance of proactive stress management support. Organizations can deploy evidence-based programs, such as structured stress management training and employee assistance programs, which have demonstrated positive average effects on strain and wellbeing outcomes (Richardson and Rothstein, 2008). Because burnout is reliably associated with turnover intentions (Lee and Ashforth, 1996; Swider and Zimmerman, 2010), systematic investments in workload norms, recovery opportunities, and flexible scheduling are likely to yield retention benefits as well as health improvements.

Finally, the moderating role of RESE suggests that talent development should prioritize enhancing emotional competence. RESE reflects perceived capability to down-regulate negative affect and express positive affect (Caprara et al., 2008), fitting within both JD–R’s personal-resources perspective (Xanthopoulou et al., 2007) and the Conservation of Resources theory (Hobfoll et al., 2018). Embedding emotional regulation micro-skills into onboarding training and continuous learning can strengthen employees’ ability to cope with social job demands such as DL, thereby supporting a healthier organizational climate and reducing turnover risk.

### 5.3 Research limitations and prospects

The cross-sectional nature of our design limits causal inference and can bias mediation estimates. To establish temporal precedence, future research should employ multi-wave longitudinal designs to measure DL, burnout, and turnover intentions across separate waves (e.g., three waves with 4–6-week lags). In addition, cross-lagged models that separate within-person from between-person variation (e.g., random-intercept cross-lagged panel models) and experience-sampling protocols can identify short-term dynamics that are missed by single-shot surveys (Maxwell and Cole, 2007; Ployhart and Vandenberg, 2010; Hamaker et al., 2015). Together, these designs increase confidence in the credibility of indirect effects and help adjudicate between alternative causal orderings.

The present study’s reliance on single-source self-reports raises concerns about common method variance (CMV). To mitigate CMV, follow-up studies should combine multi-source measures, such as supervisor or peer ratings for leadership and affective reactions, with objective indicators (e.g., HR-recorded turnover). Researchers should also introduce temporal and proximal separation between predictors and outcomes, vary scale formats, and consider diagnostic and corrective techniques (marker variables or latent-method-factor models) to assess and control method bias (Spector, 2006; Leonard et al., 2024).

Factors not observed in this study may confound the estimated relationships. Team climate, selection of specific leaders, or concurrent change initiatives could induce endogeneity. Designs that leverage team/leader fixed effects, leader-change natural experiments, or quasi-experimental interventions (e.g., civility training or anti-incivility policies rolled out in phases) can strengthen identification and reduce concerns regarding omitted variables.

The specific context of this study—China’s technology sector—may amplify social demand processes, limiting generalizability across other regions or sectors. Replications across industries and cultures will help assess generalizability and map boundary conditions. To ensure construct comparability, future work should test longitudinal and multisource measurement invariance (configural/metric/scalar), which is essential before comparing structural paths across time or sources (Vandenberg and Lance, 2000).

Finally, moderation tests are statistically demanding. Because interaction effects tend to be small and reliability loss attenuates them further, future research should plan to employ larger samples, use reliability-corrected indicators or latent variable modeling, and pre-register focal contrasts. Simulation-based power analysis is advisable for calibrating expected effect sizes and design parameters (Aguinis et al., 2005).

## 6 Conclusion

Grounded in the Job Demands–Resources and Conservation of Resources frameworks, this study used survey data gathered from Chinese technology professionals to examine whether DL is linked to TI via JB and whether regulatory emotional self-efficacy (RESE) moderates the direct DL–TI association. Analyses revealed a positive association between DL and TI, with JB partially mediating this association [indirect effect = 0.061, 95% CI (0.029, 0.088), accounting for approximately 12% of the total effect]. In addition, RESE buffered the direct DL–TI association. Simple-slope probes illustrated that the DL–TI link was weaker at high RESE (*B* = 0.165) than at low RESE (*B* = 0.485), evidencing attenuation rather than a crossover pattern. Taken together, these findings characterize DL as a chronic social demand that erodes resources and elevates burnout, while RESE functions as a personal resource that dampens the translation of DL into quitting cognitions.

Practically, organizations should minimize DL by establishing clear standards and accountability, monitoring and addressing burnout, and investing in the development of employees’ emotion-regulation efficacy. Conceptually, the results extend JD–R by foregrounding leadership as a social demand and by documenting RESE’s buffering role in the DL–TI pathway. Given the cross-sectional, single-source design, future longitudinal and multi-source investigations are necessary to corroborate the temporal ordering and to delineate boundary conditions precisely.

## Data Availability

The original contributions presented in this study are included in this article/Supplementary material, further inquiries can be directed to the corresponding author.
