# Supplementary material for: The impact of destructive leadership on turnover intention among Chinese technology professionals: the mediating role of job burnout and the moderating role of regulatory emotional self-efficacy
Source: Front Psychol. 2025 Nov 17;16:1698652. doi: 10.3389/fpsyg.2025.1698652 (PMC12665662; doi:10.3389/fpsyg.2025.1698652)
Supplement: Supplementary file 1 [file Table_1.docx]

**Appendix A. Questionnaire**

**Demographic information**

Q1. Gender

Q 2. Age

Q 3. Educational level

Q4. Work seniority

**Destructive Leadership Questionnaire**

Q5. My supervisor ridicules me.

Q6. My supervisor makes negative comments about me to others

Q7. My supervisor tells me my thoughts or feelings are stupid

Q8. My supervisor ridicules me

Q9. My supervisor tells me I’m incompetent.

**Job Burnout Questionnaire**

Q10. I feel emotionally drained from my work.

Q11 . I feel used up at the end of the workday.

Q12. I feel tired when I get up in the morning and have to face another day on the job.

Q13. Working all day is really a strain for me.

Q14. I feel burned out from my work.

Q15. I have become less interested in my work since I started this job.

Q16. I have become less enthusiastic about my work.

Q17. I doubt the significance of my work.

Q18. I have become more cynical about whether my work contributes anything.

Q19. I can effectively solve the problems that arise in my work.

Q20. I feel I am making an effective contribution to what this organization does.

Q21. In my opinion, I am good at my job.

Q22. I feel full of energy at my work.

Q23. I have accomplished many worthwhile things in this job.

Q24. At my work, I feel confident that I am effective at getting things done.

**Emotion regulation self-efficacy Questionnaire**

Q25. Express joy when good things happen to you?

Q26. Feel gratified over achieving what you setout to do?

Q27. Rejoice over your successes?

Q28. Express enjoyment freely at parties?

Q29. Keep from getting dejected when you are lonely?

Q30. Keep from getting discouraged by strong criticism?

Q31. Reduce your upset when you don’t get the appreciation you feel you deserve?

Q32. Keep from getting discouraged in the face of difficulties?

Q33. Manage negative feelings when reprimanded by your parents or significant others?

Q34. Avoid getting upset when others keep giving you a hard time?

Q35. Get over irritation quickly for wrongs you have experienced?

Q36. Avoid flying off the handle when you get angry?

**Turnover Intention Questionnaire**

Q37. I have basically never thought of leaving my current organization.

Q38. I plan to have long-term career development in this organization.

Q39. I often feel bored with my current job and want to change to a new organization.

Q40. I am likely to leave my current organization in the next six months.

**附录A. 问卷**

**人口统计信息**

问题1.性别

问题2.年龄

问题3.教育水平

问题4.工作年限

**破坏性领导问卷**

问题5.我的主管嘲笑我。

问题6.我的主管对其他人发表关于我的负面评论

问题7.我的主管告诉我我的想法或感受很愚蠢

问题8.我的主管嘲笑我

问题9.我的主管告诉我我无能。

**工作倦怠问卷**

问题10.我感到从工作中精神疲惫。

问题11.在工作日结束时，我感到筋疲力尽。

问题12.早上起床时，我感到很累，不得不面对新的一天工作。

问题13.整天工作对我来说确实是一种压力。

问题14.我因工作而感到精疲力尽。

问题15.自从我开始这份工作以来，我对我的工作变得不那么感兴趣了。

问题16.我对我的工作变得不那么热情了。

问题17.我怀疑我工作的意义。

问题18.我越来越怀疑我的工作是否有任何贡献。

问题19.我可以有效地解决工作中出现的问题。

问题20.我觉得我正在为这个组织所做的事情做出有效的贡献。

问题21.在我看来，我擅长我的工作。

问题22.我在工作中感到精力充沛。

问题23.在这份工作中，我完成了许多有价值的事情。

问题24.在我的工作中，我对自己能够有效地完成工作充满信心。

**情绪调节自我效能感问卷**

问题25.当有好事发生在你身上时,你表达快乐的能力如何？

问题26.当达到自己设置的目标时,你感受欣喜的能力如何？

问题27.面对成功时,你体验喜悦的能力如何？

问题28.在派对上自由表达的能力如何？

问题29.孤独时，你避免产生沮丧情绪的能力如何？

问题30.受到严厉批评时，你避免产生气馁情绪的能力如何？

问题31.当你没有得到你认为你应得的赞赏时，减少你的不安能力如何？

问题32.面对困难时，你使自己摆脱挫折感的能力如何？

问题33.当被父母或重要的人训斥时，处理负面情绪的能力如何？

问题34.当别人总是让你难堪时，你避免自己恼怒的能力如何？

问题35.当遇到不公平的对待时，你从愤怒的情绪中迅速恢复的能力如何？

问题36.当你生气时，你避免自己大发雷霆的能力如何？

**离职意向问卷**

问题37.我基本上从未想过离开我现在的组织。

问题38.我计划在这个组织中进行长期的职业发展。

问题39.我经常对现在的工作感到厌烦，想换一个新的组织。

问题40.我可能会在未来六个月内离开我现在的组织。
