# Supplementary material for: The impact of destructive leadership on turnover intention among Chinese technology professionals: the mediating role of job burnout and the moderating role of regulatory emotional self-efficacy
Source: Front Psychol. 2025 Nov 17;16:1698652. doi: 10.3389/fpsyg.2025.1698652 (PMC12665662; doi:10.3389/fpsyg.2025.1698652)
Supplement: Supplementary file 2 [file Table_2.docx]

## Appendix B. One-Factor Loadings by Construct

**1. Destructive Leadership**

| Item | Std. Loading |
| --- | --- |
| Q5 | 0.794 |
| Q6 | 0.817 |
| Q7 | 0.822 |
| Q8 | 0.815 |
| Q9 | 0.795 |

**2. Job Burnout**

| Item | Std. Loading |
| --- | --- |
| Q10 | 0.776 |
| Q11 | 0.74 |
| Q12 | 0.765 |
| Q13 | 0.78 |
| Q14 | 0.732 |
| Q15 | 0.735 |
| Q16 | 0.696 |
| Q17 | 0.739 |
| Q18 | 0.72 |
| Q19 | 0.752 |
| Q20 | 0.721 |
| Q21 | 0.757 |
| Q22 | 0.794 |
| Q23 | 0.75 |
| Q24 | 0.743 |

**3. Regulatory Emotional Self-Efficacy (RESE)**

| Item | Std. Loading |
| --- | --- |
| Q25 | 0.795 |
| Q26 | 0.779 |
| Q27 | 0.786 |
| Q28 | 0.791 |
| Q29 | 0.805 |
| Q30 | 0.799 |
| Q31 | 0.811 |
| Q32 | 0.793 |
| Q33 | 0.772 |
| Q34 | 0.791 |
| Q35 | 0.812 |
| Q36 | 0.785 |

**4. Turnover Intention**

| Item | Std. Loading |
| --- | --- |
| Q37 | 0.835 |
| Q38 | 0.835 |
| Q39 | 0.861 |
| Q40 | 0.839 |
